# Quantitative measurement of spatial distribution of effective refractive index induced by local electron concentration at a nano slit

**DOI:** 10.1515/nanoph-2024-0179

**Published:** 2024-06-03

**Authors:** Dae Hee Kim, Young Ho Park, Jun Hyung Park, Duy-Anh Nguyen, Hongki Yoo, Seungchel Kim, Young-Jin Kim

**Affiliations:** Department of Mechanical Engineering, Korea Advanced Institute of Science and Technology, 291 Daehak-ro, Daejeon, Republic of Korea; Department of Optics and Mechatronics Engineering, College of Nanoscience and Nanotechnology, 34996 Pusan National University, Busan 46241, Republic of Korea

**Keywords:** nano slit, effective refractive index, local electron concentration, quantitative measurement, nanophotonics, surface plasmon polariton

## Abstract

Surface plasmon polaritons (SPPs) have found their key applications in high-sensitivity biomolecular detection and integrated photonic devices for optical communication via light manipulation at nanostructures. Despite their broad utility, SPPs are known to be accompanied by other complex near-field propagation modes, such as quasi-cylindrical waves (QCWs) and composite diffracted evanescent waves (CDEWs), whose electromagnetic and quantum propagation effects have not been comprehensively understood especially regarding their mutual interaction with SPPs. In this study, we addressed this complexity by employing a nano groove structure and a high-stability broadband femtosecond laser as a light source, the spatial phase distribution around the nano slit edge was measured with relative stability of a 4.6 × 10^−11^ at an averaging time of 0.01 s. Through this spatial phase spectrum, we precisely measured the nonlinear distribution of effective refractive index changes with an amplitude of 10^−2^ refractive index units at the edge of the nano slit–groove structure. These results reveal that the near-field effects on local electron concentration induced by nanostructure’s discontinuity can be quantitatively measured, which can contribute to a deeper understanding of SPP phenomena in nanostructures for the optimal design and utilization of the SPP effects in diverse nano-plasmonic applications.

## 1 Introduction

Surface plasmon polaritons (SPPs) are associated with the coupled collective oscillations of electrons at the interface of metallic and dielectric materials. In recent years, SPPs have gained significant attention because they offer two primary characteristics. Firstly, SPP enables us to focus and propagate the light on a smaller scale than the optical diffraction limit; thus, they can be used in nanophotonic-integrated circuits [1]–[3], high sensitivity biosensor [4]–[6], and laser-induced EUV generation [7]–[9] that requires high electric field concentration. Secondly, SPP provides higher sensitivity to the local changes of geometric structural dimensions [10], [11] or refractive indices [12–16], making them invaluable in the precision measurements of physical displacements or refractive indices for chemical/biosensing [17–20]. Therefore, SPPs have facilitated interesting applications in achieving higher sensitivity measurements in various scientific and technological fields in the recent two decades.

Despite the active research efforts on SPP, a comprehensive understanding of their behavior within nanostructures still remains elusive. This difficulty arises from the coexistence of other near-field optical phenomena, such as quasi-cylindrical wave (QCW) [21–24] and composite diffracted evanescent wave (CDEW) [25–30], alongside SPP in nanostructures. QCW and CDEW describe phenomena other than SPP during the interaction of light with nanostructure and, therefore, exhibit similar characteristics. In order to distinguish SPP from QCW and CDEW, higher precision amplitude and phase spectrum should be attained by using a highly stable light source with a long coherence length and high signal-to-noise ratio (SNR). In addition, the spatial distribution of the local refractive index is a prerequisite to analyzing the discontinuous or finite characteristics of SPP at nanostructures. However, it has been difficult due to these limitations, the measurement of SPP’s phase behavior and effect of concentrated charge density still remains a challenging task.

In this study, we propose a novel quantitative amplitude/phase measurement approach that overcomes the complexity of the phenomenon by fabricating a nano slit–groove structure, allowing for the separation of QCW and CDEW from the SPP. This approach enables the precise measurement of the phase characteristics of SPP generated solely from the nano slit through the nano groove. By leveraging a broadband highly coherent mode-locked femtosecond laser as a light source to plasmonic interferometry, the SPP amplitude/phase spectrum could be successfully acquired with a high SNR. The adjustment of the carrier frequency of the signal enables the selective filtering and analysis of the desired signal, thereby significantly simplifying the signal processing procedure. For the demonstration, we experimentally acquired the phase spectrum of coherent SPP at the proposed nano slit–groove structure with a high spatial resolution of 790 nm and a 1000-times higher SNR at the high carrier frequency of interference between photon–SPP–photon by nano slit–groove and reference beam. We also observed the spatial variations of the effective refractive index at the edge of nano slit due to the local electron concentration; the maximum charge density concentration was at the nano slit by the structural discontinuity resulting in a higher electron concentration. These findings not only promise to deepen our comprehensive understanding of plasmonic nanostructures but also lay the groundwork for achieving superior results in precision optical measurement and actively manipulating the light in nanophotonic applications. We hold the conviction that this research represents a pivotal step toward the advancement of fundamental knowledge and practical applications of nano-optics and material science.

## 2 Photon–SPP–photon conversion at a nano slit–groove structure

**Figure 1:**
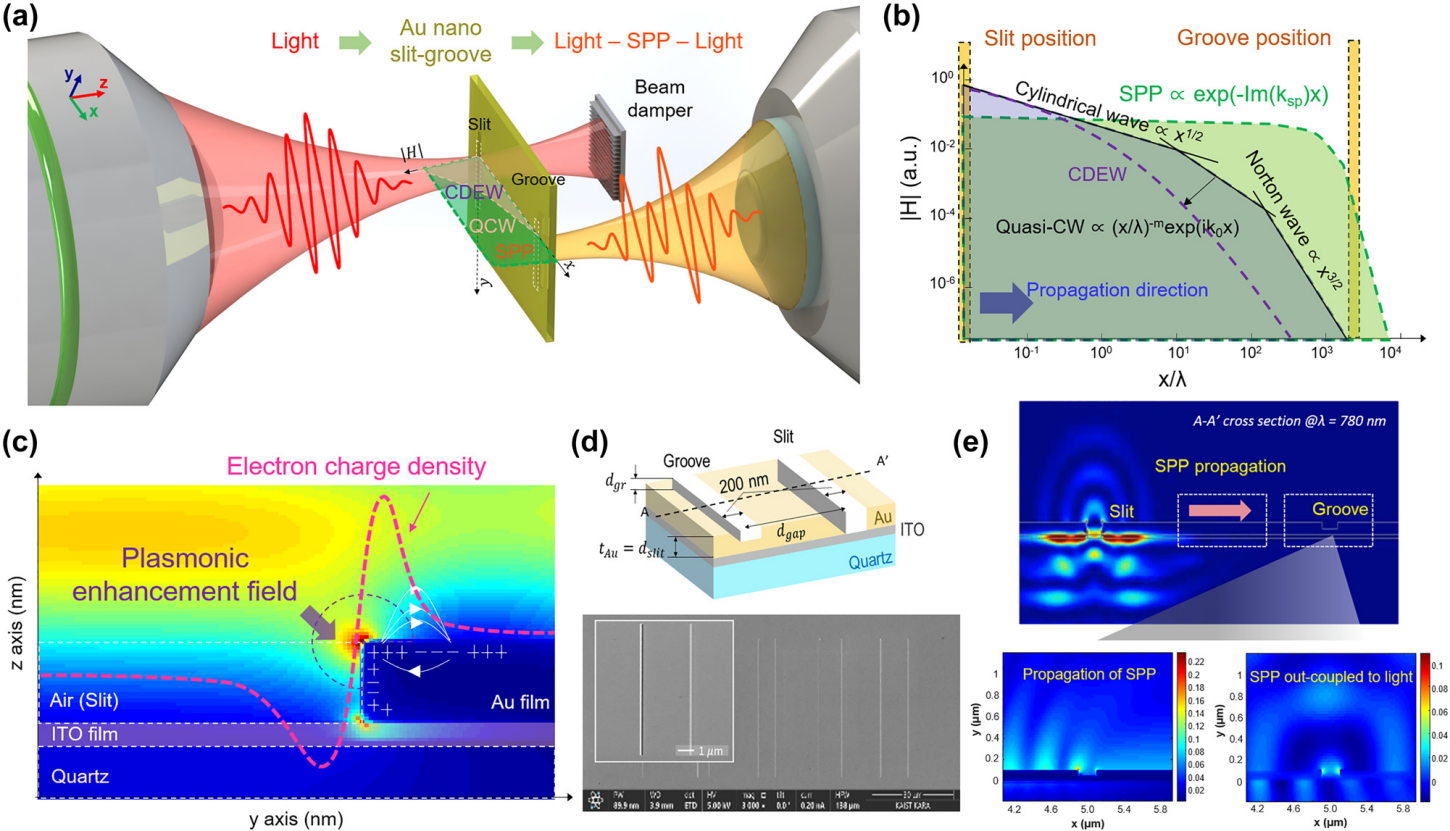
Coherent SPP excitation, propagation, and out-coupling on nano slit–groove structure. (a) Gold and air interface propagation of coherent SPP generated by femtosecond laser from nano slit and changes in phase and amplitude of the light. (b) Propagation length of magnetic field radiated on air/gold interface by vertically polarized light. (c) Plasmonic enhancement field and distribution of electron charge density of *y*–*z* cross section of the nano slit edge. (d) The nano slit–groove structure used in the experiment and its fabrication results measured by SEM. (e) Simulation results of electromagnetic wave numerical finite-difference time-domain (FDTD) of 780 nm wavelength in nano slit–groove structure. *L*, *d*_gap_, distance between slit and groove; *d*_gr_ and *d*_slit_, height of groove and slit; *t*_Au_, thickness of Au film.

Figure 1(a) represents a concept of our study for monitoring SPP only while inhibiting the presence of QCW within the nano slit. Femtosecond laser pulses, well-known for their high frequency stability and phase coherence (as demonstrated by two Nobel prizes, one regarding the frequency comb and the other concerning attosecond pulse generation), are directed onto the nano slit. The pulses are coupled to the air–gold interface, where the SPP is generated and propagate over the gold surface. It’s worth noting that although SPP, QCW, and CDEW are excited at the nano slit, their propagation lengths significantly differ, as shown in Figure 1(b). QCW’s propagation length exhibits typically in the range of a few micrometers (4 μm, at *λ* = 780 nm and *m* = 0.8 in the gold film) [21], [31], while SPP extends over much longer distances, well beyond tens of micrometers. CDEW is known to be a creeping wave with attenuation scaling greater than 1/*x* ^1/2^ as a function of distance *x* from the linear nano-object in the visible light and have a propagation distance on the around 3 μm for a 780 nm wavelength [25], [26]. To completely suppress QCW and CDEW, and capture only the SPP, the experiments were conducted with a 10-μm slit–groove separation distance, which significantly exceeds the propagation length of QCW and CDEW. The width and length of the nano groove and slit were 200 nm and 40 μm, respectively, so that SPP was generated in the central wavelength (*λ* = 780 nm) of the femtosecond laser. As the SPP propagates at the air and gold interface, they undergo substantial changes in both amplitude and phase, which is subsequently emitted from the nano groove. This to be QCW and CDEW suppression allows us to measure the out-coupled light merely from SPPs. Now, it is worth emphasizing that, the frequency comb of a femtosecond pulse laser demonstrated to retain their properties under surface plasmon resonance (SPR) phenomena within nanostructures with high fidelity [32]. The interaction of the frequency comb with the nanostructures produces coherent SPPs, which facilitates the in-depth study of the spatial and spectral changes in SPP’s amplitude and phase propagation. Figure 1(c) shows a *y*–*z* cross-sectional view of the amplitude of electric field at the nano slit edge side. Specifically, at the nano slit, the geometric discontinuity generates an extensive plasmonic enhancement field compared to the inside of the nano slit; this is due to a significant change in the electron charge density relative to the inside part, which can be observed through the refractive index distribution therearound.

As depicted in Figure 1(d), we fabricated the nanostructures on a quartz substrate with a 500-μm thickness. A 25-nm-thick adhesion layer of indium tin oxide (ITO) was deposited on the quartz substrate by magnetron DC (direct current) sputtering. Subsequently, a 100-nm-thick layer of gold (Au) was subsequently sputter-deposited on top of the ITO layer via the same process. To achieve the desired nano slit–groove structures, a focused ion beam (FIB) process was employed, which precisely machined the slit–groove distance to 10 μm. The height of the slit (*d*_slit_) and groove (*d*_gr_) were maintained at 100 nm and 50 nm, respectively, while the width of the groove and slit were set to be the same to 200 nm. The patterned nanostructures were confirmed using scanning electron microscopy (SEM). For the optimal SPP generation and propagation, the height and width of slit and groove were designed based on numerical finite-difference time-domain (FDTD) simulation software, guided by Equation (1) across the spectral range of 550–850 nm.

(1) $$k_{\mathrm{spp}}=k_{0}\sqrt{\frac{\varepsilon_{m}\varepsilon_{d}}{\varepsilon_{m}+\varepsilon_{d}}}$$

The simulation results in Figure 1(e) indicates that the electromagnetic field is excited at the nano slit and propagates along the Au surface, at the wavelength of 780 nm. We also confirmed that the generated SPPs can be efficiently out-coupled from the nano groove back to photons. Nevertheless, a significant decrease in intensity of more than 50,000 times was observed between the incident beam and the beam that underwent the photon–SPP–photon conversion process. Most of the light intensity loss occurs when passing through the nano slit (mostly reflection), and only wavelengths that satisfy the plasmon resonance equation in the transmitted beam are converted into surface plasmon polaritons. Thereafter, there is some loss as it propagates along the gold surface. Taking this important intensity difference into account, achieving high coherence and high SNR in the experimental system is the prerequisite; this necessity highlights the importance of our proposed approach in effectively capturing and characterization of the behavior of SPP at the nanostructures.

## 3 High coherence and high SNR phase spectroscopy with a high lateral resolution

**Figure 2:**
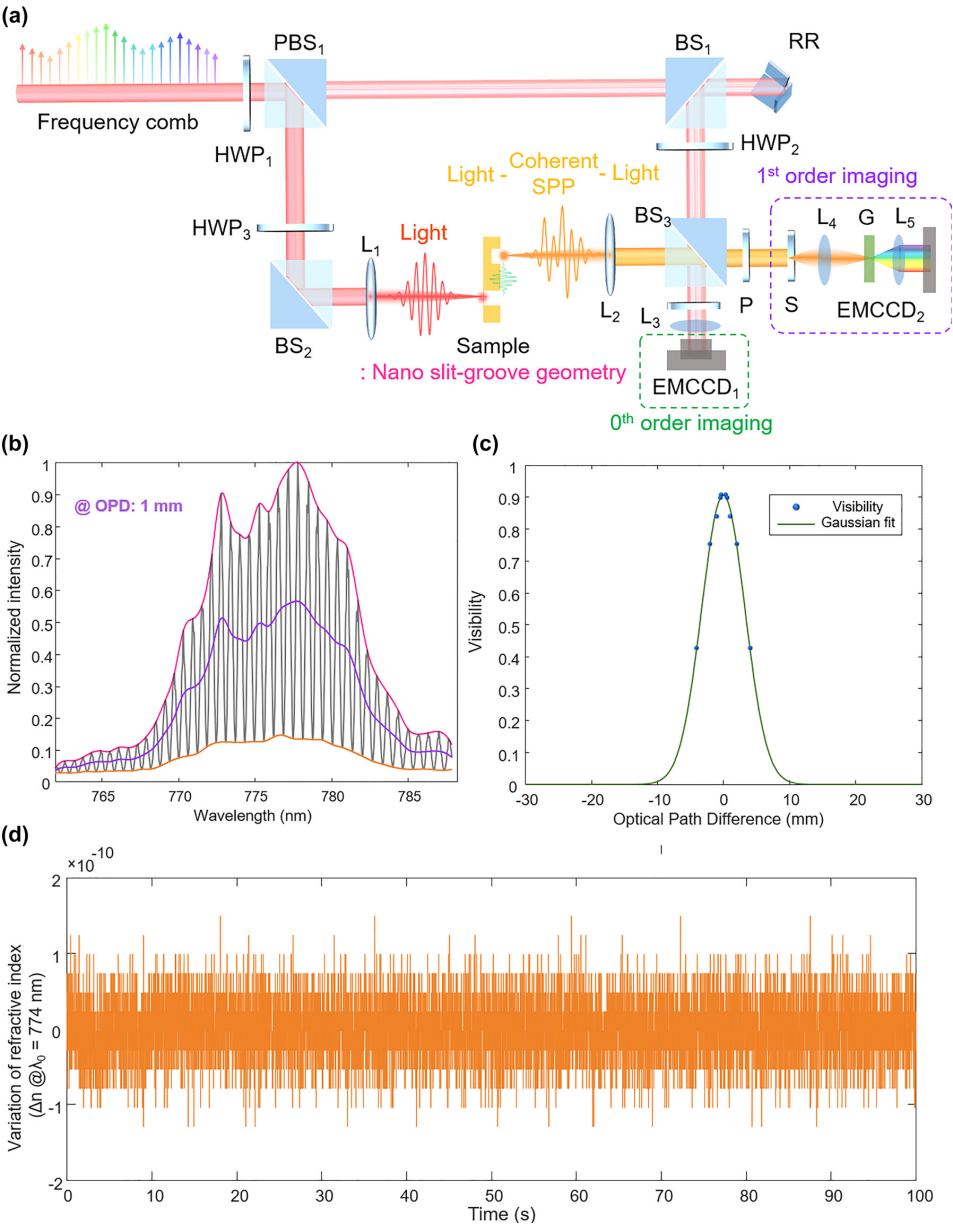
Polarization-dependent Mach–Zehnder interferometer for coherent and high SNR phase spectroscopy. (a) The system facilitates the measurement of coherent SPP generated from gold nano slit–groove structure. The intensity ratio of the measurement light and reference light can be controlled using a half-wave plate and a polarization beam splitter. The optical path difference (OPD) can be adjusted by moving the retroreflector in the reference light path. A charged coupled device (CCD) and an imaging spectrometer used as detectors for acquiring the 0th and 1st order images, respectively, enabling precise alignment and extraction of the phase spectrum of SPP’s propagation. (b) The spectral interferogram exhibits high visibility even at a large OPD of 1 mm. (c) The visibility of the spectral interferogram is analyzed with varying OPD to evaluate the coherence of the measurement system and the linewidth of the spectrometer. (d) The refractive index change is calculated based on the phase slope derived from 10,000 consecutive measurements of the spectral interferogram at an OPD of 1 mm. HWP, half-wave plate; PBS, polarization beam splitter; BS, beam splitter; RR, retroreflector; L, lens; P, polarizer; EMCCD, electron multiplying charged coupled device; S, slit; G, grating component.

Figure 2(a) depicts the experimental setup of a high-precision, frequency-comb–referenced phase spectroscopy system that provides wide tunability in coherence and SNR. The system utilizes a femtosecond pulse laser with a center wavelength of 780 nm, the repetition rate of 83.33 MHz, and the pulse duration of 150 fs (Toptica, FemtoFiber Pro NIR). By using a frequency-comb with high coherence, the light that has undergone light–SPP–light conversion can interfere with the reference light, and the carrier of the interference pattern can be adjusted over a wide range for easy signal processing. In addition, because the intensity of measurement light becomes very weak, a system with high SNR that can obtain interference signals with high visibility by adjusting the intensity of reference light and measurement light is essentially needed. Therefore, the measurement system is based on a polarization-sensitive Mach–Zehnder interferometer, which allows for the adjustment of the intensities of the measurement and reference beams by a large unbalance ratio using a half-wave plate (HWP_1_) and a polarization beam splitter (PBS). Subsequently, the reference beam passes through a beam splitter (BS_2_) and a retroreflector (RR) before reaching the detector via the final BS_3_. Meanwhile, the measurement beam is focused onto the nano slit, the incident part of the nano slit–groove structure, by a focusing lens. The coherent SPP generated from the slit propagate and are out-coupled as the form of photons at the nano groove. They then enter the detector after passing through the subsequent lens and BS. A high numerical aperture (NA) objective lens with a magnification of ×20 is employed for light coupling and imaging. The detector system consists of an imaging spectrometer and a high-sensitivity electron multiplying charge-coupled device (EMCCD (Andor, iXon Ultra 888)), which acquires the sample images at 0th order diffracted beam and sample spectrum at the focal spot at the 1st order diffracted one. These two detect mode (0th and 1st orders) enable the elimination of unexpected dispersion effects due to the tilt and imaging position adjustment; the resulting simultaneous acquisition of spatial distribution of the light intensity and spectral information allows for comprehensive understanding and analysis of the plasmonic samples.

To assess the coherence degree of the measurement system, we examined the visibility of the spectral interferogram of a single spatial location from the spatial 1st order image in the absence of the nano slit–groove sample, as shown in Figure 2(b) and (c). The OPD was adjusted by varying the distance between BS_2_ and the retroreflector in the reference path; the visibility of 0.7 or higher was acquired even at long OPDs. Analyzing the full-width at half-maximum (FWHM) of the Gaussian fit from Figure 2(c), we determined the coherence length to be 7.0176 mm, which implies the linewidth of 0.027 nm (Δ*υ* = 0.136 THz) per CCD pixel. The calculated wavelength interval was 0.025 nm (Δ*υ* = 0.127 THz) based on the grating and CCD’s installation parameters, which closely matched the experimental value. Furthermore, in order to evaluate the system stability, we applied the spectrally resolved interferometer [33]–[35] principle to obtain the phase slope (d*φ*/d*ν* = 2π/cnL) from the spectral interferogram measured at a 1-mm OPD, as shown in Figure 2(d). The refractive index variation (∆*n*) was recorded at a 100-Hz update rate over 10,000 samples, yielding a deviation of 4.6861 × 10^−11^, which confirms a sufficiently stable system and environment even at an intentionally set 1-mm long OPD.

**Figure 3:**
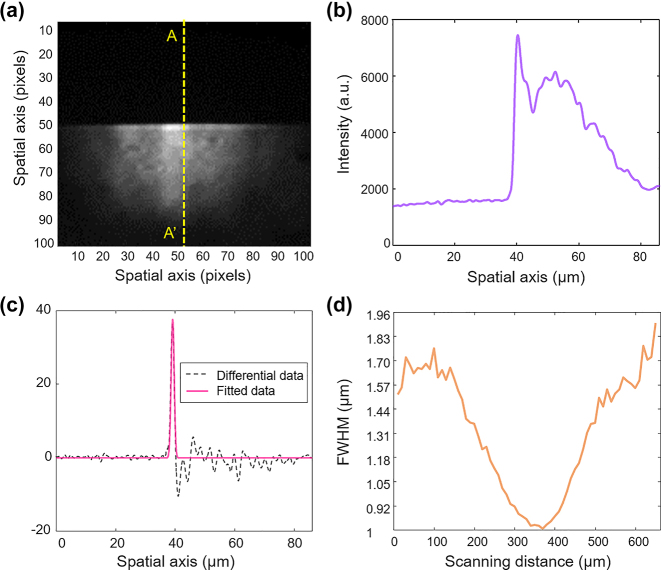
Evaluation of lateral resolution. By assessing the full width at half maximum (FWHM) of the intensity derivative with respect to the position of the sample blocking half of the light. (a) Image of the sample blocking half of the light. (b) Intensity profile along the A–A′ cross section of (a). (c) Derivative graph and Gaussian fitting of (b). (d) FWHM values fitted with Gaussian along the sample scanning positions.

Figure 3 shows the process of evaluating lateral resolution. To achieve a high lateral resolution, a set of high-NA ×20 objective lenses was utilized. Figure 3(a) represents the 0th order image obtained by blocking the light from the measurement path; a block sample was designed and installed to obstruct the upper half of the light transmission in the reference path. Figure 3(b) illustrates a vertical cross-sectional intensity profile of Figure 3(a), showing a sharp increase of the intensity around the central position, followed by the intensity distribution to the right end. By taking the numerical derivative of Figure 3(b), the resulting data shown in Figure 3(c) and its Gaussian fit enable us to set the lateral resolution. The lateral resolution was determined to be 790 nm by taking the FWHM of the transmitted intensity while scanning the sample position in the measurement beam. Considering the 16 μm × 16 μm pixel size of the EMCCD, the calculated lateral resolution was expected to be 800 nm; this slight discrepancy in resolution is expected to be introduced by the relay lens inserted in between. This experiment on the lateral resolution using the 0th order imaging also enabled higher precision image positioning.

## 4 Phase spectrum analysis of coherent SPP propagation

**Figure 4:**
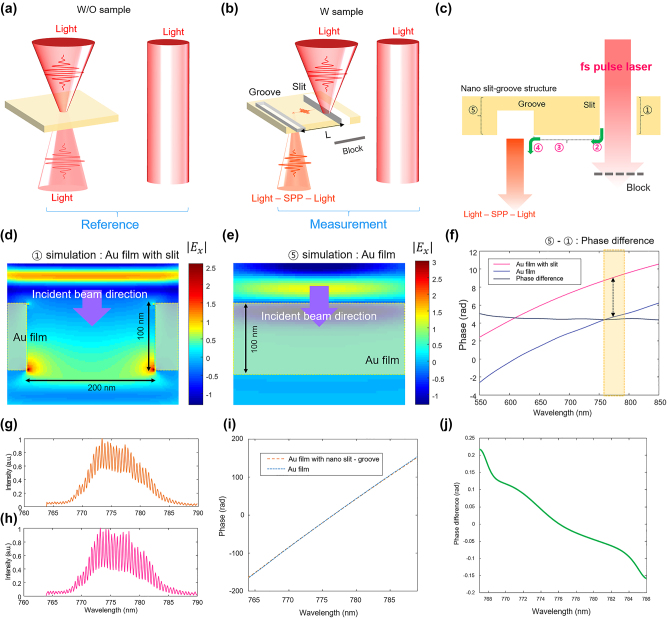
Extraction of phase spectrum of coherent SPP propagation generated from the nano slit. (a) and (b) Conceptual methods for acquiring the measurement and reference interference signals for extracting the phase spectrum of SPP’s propagation. (c) Diagram of elements of phase variation generated in the gold nano slit–groove structure. (d) and (e) Numerical finite-difference time-domain (FDTD) simulation results of electromagnetic field at a wavelength of 780 nm for the corresponding gold slit (①) and gold film transmission (⑤) in (c). (f) Phase variations according to the wavelength generated during the transmission of the gold nano slit and the gold film. (g) and (h) Spectral interferogram obtained from the measured and reference signals in (a) and (b), respectively. (i) Phase slope extracted through the spectrally resolved principle from (g) and (h). (j) Phase spectrum difference obtained by subtracting two phase spectrums.

The phase spectrums were extracted from reference and measurement spectral interferograms as described in Figure 2; the reference interferogram was obtained with the nanostructure (see Figure 4(a)), while the measurement one was obtained with the nanostructures (see Figure 4(b)). During the measurement data acquisition, the imaging position of the 1st-order detector was translated by the distance corresponding to the 10-μm gap between the nano slit and the groove, ensuring measurements with the same optical path difference. Consequently, the phase difference between the measurement and the reference signal represents the phase attributed to components ① − ⑤ + ② + ③ + ④, as depicted in Figure 4(c).

The phase changes occurring during the transformation of light to SPP (photon to SPP) in the gold nano slit and groove (② & ④) result in a phase shift of π/2 [36]. Figure 4(d) and (e) display the electromagnetic field simulation results at a wavelength of 780 nm for the gold film (⑤) and gold slit (①) transmission, respectively. The simulation in Figure 4(f) confirms that their phase difference at 780 nm was 4 rad (⑤ − ①). Figure 4(g) and (h) show the experimentally attained spectral interferogram with and without the nanostructures. Through a series of data processing (Fourier transform, spectral filtering, spectral centering, and inverse Fourier transform), we can obtain the phase spectrum as shown in Figure 4(i); the resulting phase spectrum shows a highly linear slope proportional to the optical path difference. The phase spectrum due to plasmonic phenomenon at nano slit–groove can be obtained from the phase spectrum difference (Δ*φ*(*υ*)) acquired by subtracting two phase spectrums, as shown in Figure 4(j).

## 5 Spatial distribution of effective refractive index at the edge side of a nano slit

**Figure 5:**
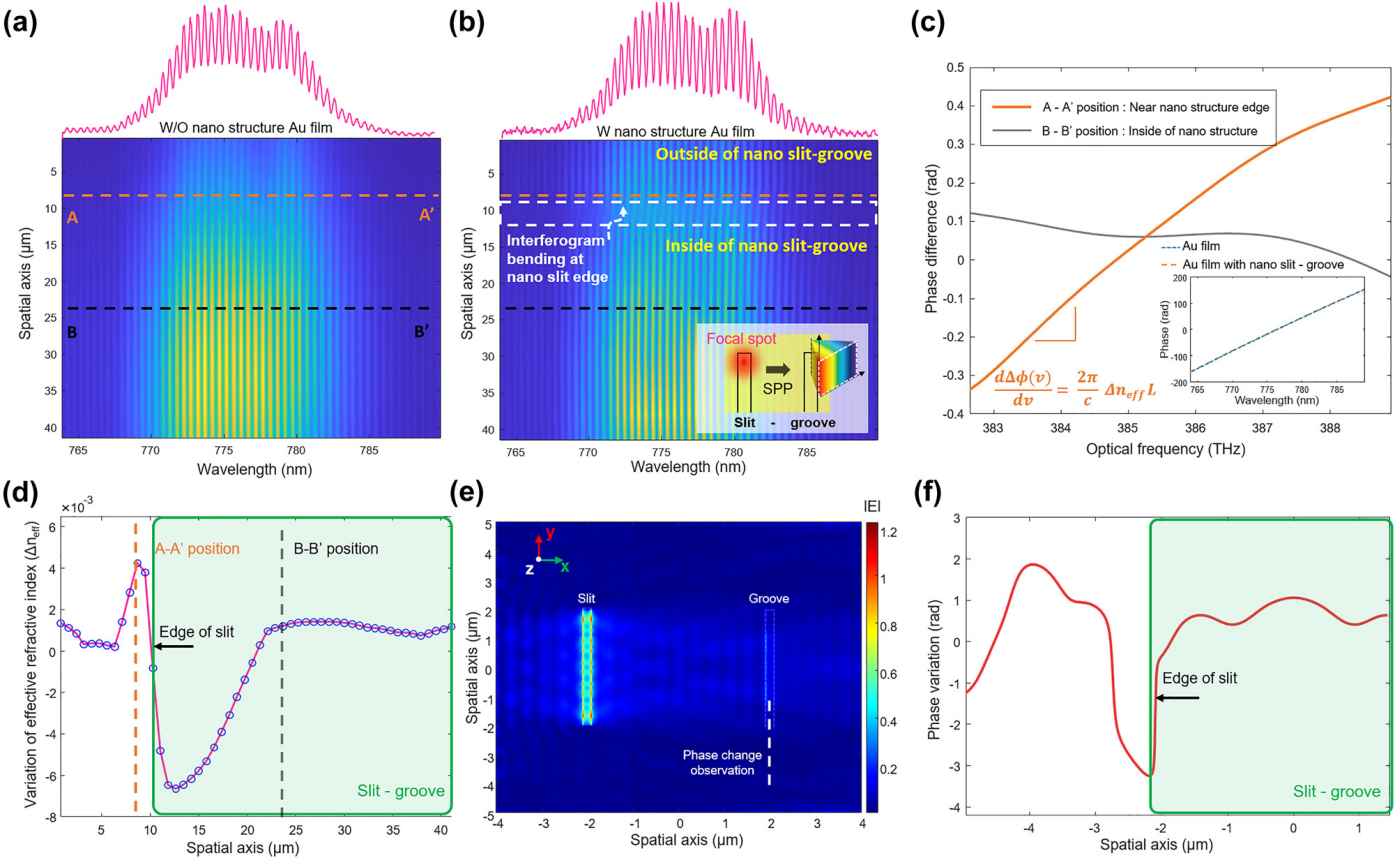
Spatial distribution of effective refractive index changes at the edge of the nano slit. (a) Interference pattern in the Au film without nanostructure. (b) Interference pattern at the edge of the nanostructure. (c) Slope of each phase spectrum difference at points A–A′ (outside the nanostructure) and point B–B′ (inside the nanostructure) in (a) and (b), respectively. (d) Spatial distribution of effective refractive index change by calculating the slope of the phase spectrum difference. (e) and (f) Numerical FDTD simulation result of phase variation and intensity of electric field on nano slit–groove structure, respectively.

Figure 5(a) and (b) depict the 1st-order spatial–spectral (vertical–horizontal) images of the reference interferogram (without nano slit–groove) and the measurement one (with nano slit–groove) on the Au thin film, respectively. The measurement interferogram (see in the upper inset of Figure 5(b)) comes out from the end of the groove where the photons are out-coupled, as illustrated in the inset figure. In Figure 5(b), the position at 10 μm represents the very edge of the nanostructure. There is only a gold film without any nanostructures below this edge line, whereas there are nanostructures above this edge. Within the white dotted region over 7–13 μm near the edge of the nano slit–groove location, the spectral interference appears to be gently sloped due to the significant phase change.

By subtracting the phase spectrum of the reference beam from that of the measurement beam at each spatial point, we could obtain the plasmonic spectral phase change (Δ*φ*(*υ*)) as shown in Figure 5(c). Here, the slope of the spectral phase change represents the change in effective refractive index, which can be described by:

(2) $$\frac{d\varphi_{\text{ref}}\left(\upsilon \right)}{d\upsilon }-\frac{d\varphi_{\text{mea}}\left(\upsilon \right)}{d\upsilon }=\frac{d\Delta \varphi \left(\upsilon \right)}{d\upsilon }=\frac{2\pi }{c_{0}}\cdot \Delta n_{\text{eff}}\cdot L$$

The gray gentle phase spectral change in Figure 5(c) corresponds to A–A′ position (inside of the nanostructure) obtained from spectral interferences in Figure 5(a) and (b), while the orange phase spectral change shows the notably steep phase slope near the edge (B–B′ position in Figure 5(a) and (b)) of nanostructure.

This observation confirms that a substantial change in effective refractive index exists near the nanostructure’s edge. The local nonlinear effective refractive index distribution near the nanostructure’s edge could be effectively plotted as shown in Figure 5(d) with the order of 10^−2^ RIU by monitoring the slope of the spectral phase change. Remarkably, a negative refractive index change was observed inside the edge of the nanostructure, which was transitioned to prominent positive values immediately outside. This interesting observation attributes to the discontinuity of the nanostructure; the nanostructure’s discontinuity results in a significantly higher charge density right outside the nano slit, contrasting the relatively lower charge concentration within right inside the nano slit, as shown in Figure 1(c). Moreover, this effect is accentuated by the presence of discontinuities not only in the nano slit but also in the nano groove. The spatial variation in electron density induces a corresponding phase change, which results in distinct bending of the spectral interference at the edge of nanostructure, as shown in Figure 5(b). Figure 5(e) illustrates the FDTD simulation results at the nano slit–groove configuration. The enhanced electric field amplitude (|E|), particularly stronger near the edge of the nano slit, is also attributed to the discontinuity effect of the nanostructure. This observation validates the formation of a significantly enhanced plasmonic field due to the locally concentrated electron density. Furthermore, Figure 5(f) shows a phase change at a position 5.0 μm above the nanostructure near the edge of the nano groove along the *z*-axis from Figure 5(e). A rapid phase change of 3.19 radians was observed at near the edge of the nano groove position (*x* = −2 μm) due to spatial electron density change. Similar to Figure 5(d), a negative phase change was observed near the internal part of the nanostructure due to the high charge density, while a slight positive phase change was observed near the external part of the nanostructure.

## 6 Conclusions

We have demonstrated the measurement of phase spectrum of SPP generated in a nano slit with a relative stability of 10^−11^, a high lateral resolution of 790 nm and high SNR by employing a nano groove structure, and mode-locked near-infrared femtosecond laser. From the spatial phase spectrum, we extracted the spatial distribution of effective refractive index change. A nonlinear distribution of effective refractive index changes with 10^−2^ RIU was observed at the nanostructure’s edge. This variation is attributed to the concentrated charge density at the discontinuous position of the nano slit. Our results lay the groundwork for proposing innovative nanostructures that can provide higher resolution and efficiency across diverse applications, thereby influencing the landscape of nanotechnology in fields such as precision measurement, sensing, and communication.
